# Brucellosis an Unusual presentation as isolated septic mono-arthritis of the knee joint: A case report

**DOI:** 10.1016/j.heliyon.2024.e26612

**Published:** 2024-02-17

**Authors:** Muad Abdi Hassan, Fatima Noor, Aram Salehi, Bassem Al Hariri

**Affiliations:** a Department of Medicine, Hamad Medical Corporation, Doha, Qatar; b Medical Education Department, Hamad Medical Corporation, Doha, Qatar; c College of Medicine, Qatar University, Qatar

**Keywords:** Brucellosis, Septic knee arthritis, Osteoarticular involvement, Brucella melitensis

## Abstract

Brucellosis is a zoonotic infection that is widely spread across the world. It is becoming more common in Middle Eastern countries such as Qatar, Saudi Arabia, and the Mediterranean region. Despite this, we need to remain vigilant as it is still prevalent in many parts of the world. The most common presentation is musculoskeletal, but it can also present as septic arthritis in the sacroiliac, hip, or knee joints. Brucella melitensis was only found in one extended culture of synovial fluid. Treatment involved a combination of antimicrobial therapy using gentamycin, doxycycline, and rifampin. A high level of suspicion for brucellosis is necessary for any patient coming from an endemic region with non-specific and chronic arthritis to ensure early diagnosis and treatment. In this case, we present a 28-year-old male who was diagnosed with Brucellosis after developing acute septic arthritis.

## 1 Introduction

Brucellosis is a zoonotic disease that is endemic in certain regions such as the Middle East and the Mediterranean. It has been increasingly prevalent in recent years, due to the consumption of non-pasteurized milk and milk products from camels, which are widely consumed in these regions. This systemic infectious disease has a wide range of clinical presentations, but it is typically characterized by a single swollen and painful joint, which is known as an osteoarticular manifestation. The most common forms of this disease are sacroiliitis and arthritis of a major peripheral joint, such as the knee, hip, or shoulder. It is important to consider brucellosis in all people with septic arthritis who reside in or travel through endemic regions [1,2].

### 1.1 Case presentation

A 28-year-old man, previously healthy with no significant past medical history or co-morbidities from Bangladesh recently came to Qatar. He was hospitalized due to swelling and pain in his right knee. He did not have a fever, but had been experiencing the pain for 7 days and had taken NSAIDs, which did not provide relief. On the third day, joint swelling and pain developed, and he experienced difficulty walking due to movement restrictions in the right knee joint. There was no confirmed history of animal contact, camel milk consumption, trauma, or skin rash. Additionally, no other joint was affected. Upon examination, he was found to be afebrile and normotensive. The right knee was tender, hot, and swollen, with a reduced range of movement. Thus, some differential diagnoses for those manifestations were septic arthritis and crystal-induced arthritis.

**Fig. 1:**
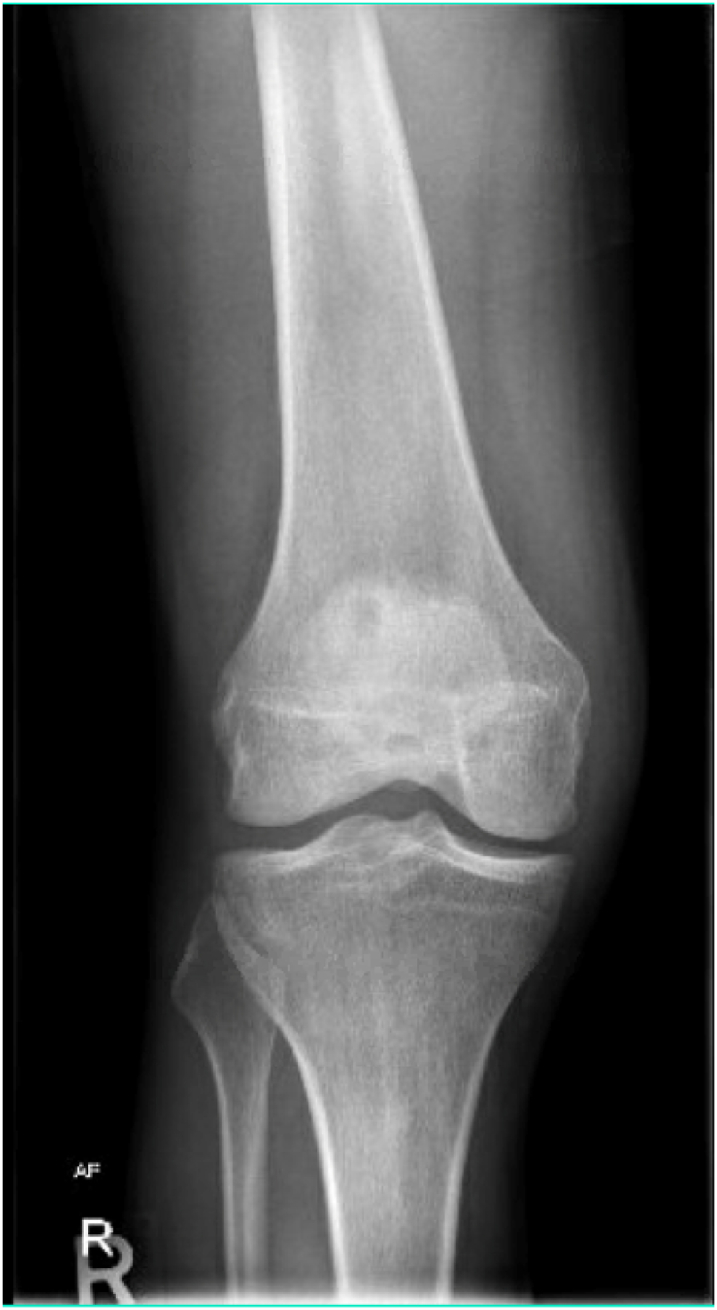
Right knee AP view normal x-ray.

**Fig. 2:**
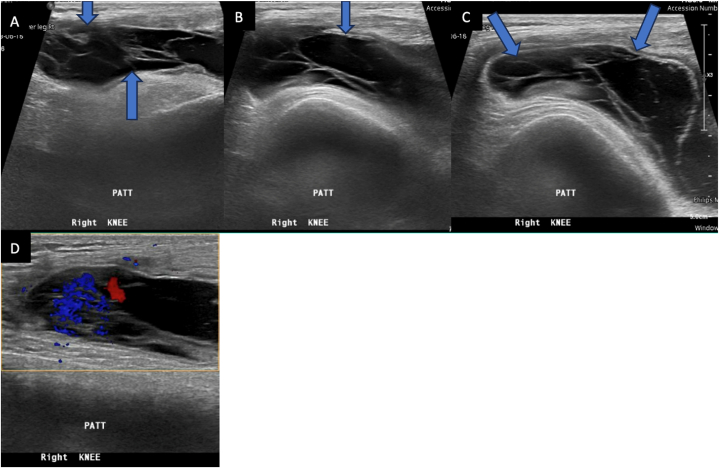
A, B, C & D: Massive right knee effusion informs of the distended suprapatellar bursa that shows internal septation with minimal peripheral hyperemia noted.

**Fig. 3:**
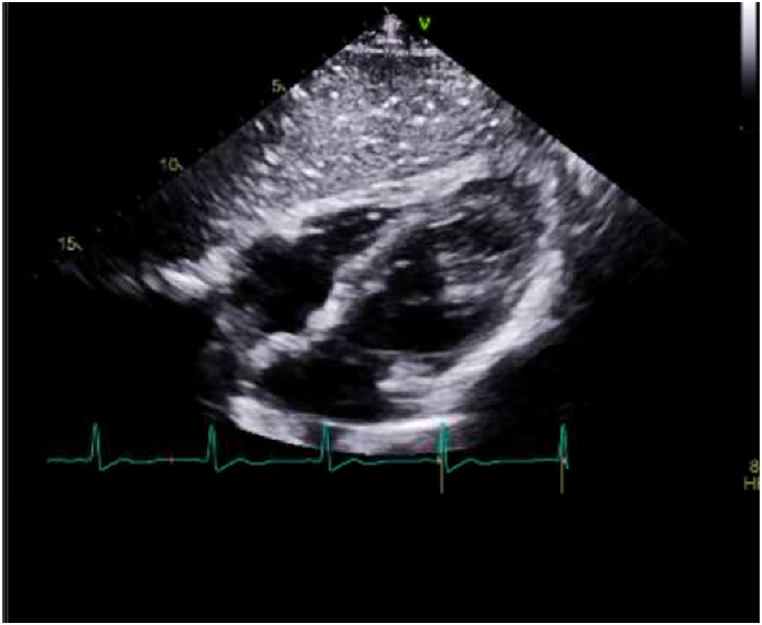
Normal echocardiogram.

The x-ray of his right knee in the AP view showed no abnormalities and was normal (Fig. 1). Soft tissue ultrasound of the right knee showed massive knee joint effusion with septation (Fig. 2A–D) The echocardiogram showed no vegetation with an ejection fraction (EF) of around 57% to exclude infective endocarditis (Fig. 3). Blood tests for the detection of autoimmune markers including the rheumatoid factors revealed no autoimmune disorders associated with the patient. Also came negative for Treponemal and Neisseria species (Table 1).

**Table 1:** (Laboratory tests).

Test	Admission	Discharge after 7 days	Normal Reference Ranges
WBC	7.8*10^3^/uL	7.3*10^3^/uL	4-10*10^3^/uL
RBC	5.3*10^6^/uL	5.2*10^6^/uL	4.5–5.5*10^6^/uL
Haemoglobin	14.4 gm/dl	14.0 gm/dl	13-17 gm/dl
HCT	42.4%	41.6%	40–50%
MCV	79.8fl	79.5fl	83-101fl
MCH	27.1pg	26.8pg	27–32pg
MCHC	34.0 gm/dl	33.7 gm/dl	31-34 gm/dl
Platelet	189*10^3^/uL	330*10^3^/uL	150-410*10^3^/uL
Neutrophil	72.1%	55.5%	40–69%
Lymphocyte	15.1%	25.6%	30–40%
Eosinophil	2.3%	3.5%	<1%
ESR	24mm/hr	24mm/hr	2–28mm/hr
CRP	175.6mg/dl	68.2mg/dl	0–5mg/dl
Prothrombin Time	–	13.7 seconds	9.4–12.5seconds
INR	–	1.2	1
APTT	–	32.3 seconds	25.1–36.5seconds

Additionally, the blood test showed some abnormalities, including abnormal indexes related to iron deficiency anemia, despite normal levels of hemoglobin. The Brucella serology test was positive and the synovial fluid aspirate test showed some abnormalities, as seen in Table 2, Table 3. The culture detected Gram-negative coccobacilli profuse polymorphonuclear leukocytes from enrichment medium Brucella species, and Brucella melitensis was detected by PCR in synovial fluid Table 3. It is important to note that Brucella belongs to the WHO risk group. Finally, a diagnosis of isolated septic mono-arthritis due to brucella melitensis was made. The patient was given gentamycin at a dose of 5 mg/kg once through an intravenous injection for seven days. Additionally, he was prescribed doxycycline at a dose of 100 mg and rifampin at a dose of 450 mg twice daily for twelve weeks. Upon discharge, the patient was advised to avoid consuming unpasteurized dairy products and was scheduled for a follow-up appointment in 5 weeks.

**Table 2:** (Synovial Fluid)Normal values.

Appearance	Turbid	Transparent
Color	Light Yellow	Clear
Lactic acid	12.8	<2.5
TNC	18,500	<200
RBC	2250	<2000
Neutrophil	55.0	<20
Lymphocyte	18.0	<15
Monocyte	22.0	<65

**Table 3:** (bacterial/parasite serology).

Brucella abortus Titer	Positive 1:160
Brucella melitensis Titer	Positive 1:320
Brucella Ab IgG	Positive
Brucella Ab IgM	Positive
Treponema Pallidum Ab	Non-Reactive

## 2 Discussion

Brucellosis is caused by an intracellular bacterium, with Brucella melitensis as the most common species. The old name of Brucellosis was ‘Malta fever’ as it had its origin in the Mediterranean area. The most common routes are occupational contacts like veterinary, animal husbandry, or consumption of contaminated milk products. A case of human-to-human transmission from a mother to a child through transplacental or perinatal exposure has been reported [1,3] Pathogens like bacteria can be used as a tool for bioterrorism, Brucella can infect through inhalation, potentially it can be used as a bioweapon [4]. There is a high incidence of brucellosis in the Middle East, Central Africa, and North Africa, more than 100/100,000 person-years with the highest rate among aged 40–49 years and the lowest rate among children less than 10 years 2.9/100,000. A study showed that there were more cases in families who had animals including camels, sheep, goats, and cows [[5], [6], [7]].

Brucellosis is a bacterial infection that can cause a variety of symptoms, including fever, chills, sweating, loss of appetite, weight loss, lymph node swelling, and enlargement of the liver and spleen. The disease often affects the musculoskeletal system, but its presentation can be non-specific and misleading. It can sometimes lead to joint and bone infections such as spondylitis, bursitis, sacroiliitis, peripheral arthritis, and osteomyelitis. Joint involvement is most common in the sacroiliac, knee, and hip joints, followed by the spine. These symptoms can occur as a complication of the systemic disease, or they can occur in isolation from other systemic manifestations. The most common involvement of joints was seen in the sacroiliac (26%), knee (25%), and hip (18%) followed by spine (8%) [8].

Brucellosis is diagnosed by conducting a serum agglutination test (SAT) to detect rising antibody titers to brucella antigens. This test is positive in most endemic areas. However, diagnosing brucellosis arthritis in non-endemic areas can be challenging. In such cases, cultures are taken from blood tissue and bone marrow. Blood cultures are positive in 53.4%–90% of cases of systemic brucellosis, but this decreases over time. A study by Shehabi et al. found that blood cultures have a sensitivity of 44.4% compared to 27.7% for marrow cultures. However, the sensitivity of blood cultures has increased up to 95% with an incubation time of only 7 days [9,10]. Automated systems, such as the BACTEC system and the Isolator Microtubule system, are used to detect the presence of Brucella melitensis. These systems have shown 100% and 78.6% sensitivity, respectively. It is important to note that blood cultures can be negative in cases of single-joint disease, while positive body fluid can be present. Therefore, laboratory investigations are crucial in diagnosing brucellosis arthritis. Positive blood and synovial cultures can co-exist, but synovial fluid analysis can help rule out crystal-induced and viral arthropathies. Leucocyte counts in synovial fluid can vary widely in different patients, ranging from high to low [[11], [12], [13]].

In addition to the conventional methods, ELISA is also a serological test but has less sensitivity and specificity. PCR assay and PCR- RFLP (restriction fragment length polymorphism) is a molecular method and if available has high sensitivity and specificity. It can be used for epidemiological, taxonomic, evolutionary, and diagnostic purposes [8]. In our case, we used the serological test PCR and the synovial fluid culture, both were satisfied to detect the species and the strain type in it. According to a systemic review that included 30 randomized control trials and 77 treatment arms, the treatment for brucellosis, showed that monotherapy had two times overall failure than combined treatment when given for the same duration (relative risk 2.56, confidence interval 1.55 to 4.23 in 5 trials). Dual therapy with doxycycline-rifampicin had a higher failure rate than doxycycline-streptomycin (RR 2.80, 95% CI 1.81 to 4.36 in 13 trials). Gentamicin compared to streptomycin in combination with tetracycline showed high rates of failure and relapse with streptomycin (1.45,0.52 to 4.00 for failure in 2 trials), side effects of gentamicin were less common (0.19,0.01 to 3.99). Tetracycline/Streptomycin was superior to tetracycline/rifampicin. One trial assessed a combination of co-trimaoxazole with rifampicin and doxycycline, showing high failure and relapse without doxycycline (2.86,1.25 to 6.54). Quinolones/rifampicin proved to be less effective than doxycycline with streptomycin or rifampicin (1.83, 1.11 to 3.02 for failure in 5 trials), but quinolones are not currently recommended. Combination of Triple regimen (Doxycycline-rifampicin) when combined with aminoglycoside had better effectiveness (2.50, 1.26 to 5.00 in 2 trials). Doxycycline 100 mg twice daily, gentamicin 240 mg once daily, and rifampicin 900 mg once daily. Treatment given for six weeks or more proved to be effective. Treatment should be prolonged to 3 months for serious cases of brucellosis to prevent relapse [13]. In our case, was treated with triple therapy as the monotherapy or dual showed some element of treatment failure with this systematic review [13].

To differentiate septic arthritis by pyogenic organisms, from Brucellosis of the joint by brucella melitensis, the joint has moderate swelling and tenderness but erythema is not significant [14]. The diagnosis of local inflammatory disease of joints solely based on history is very challenging unless combined with other investigations to reach a diagnosis.

## 3 Conclusion

Brucellosis is a challenging problem due to its variable rheumatologic manifestations, which can mimic different types of arthritis. Clinical vigilance and thorough differential diagnoses are critical to avoid diagnostic errors and ensure appropriate care.

In non-endemic areas, a detailed history of occupation, animal exposure, and contact history is required to get a clue to the diagnosis. In addition to history, investigations like blood, synovial fluid culture, and brucella serology are performed. Antibiotics and dual or triple therapy for an adequate length of at least 6 weeks or more are required to prevent treatment failure or recurrence. Moreover, screening for Brucellosis should be done in the Brucellosis prevalent and endemic areas for all the patients who present with septic arthritis.

## Human Ethics

We obtained written informed consent from the patient to publish this case report by the journal's consent policy.

## Ethics approval and consent to participate

The article describes a case report. Therefore, no additional permission from our Ethics Committee was required.

## Funding

This case report was not funded.

## Data availability statement

This is a case report, and the data is included in the article/supplementary material/referenced in the article.

## CRediT authorship contribution statement

**Muad Abdi Hassan:** Writing – review & editing, Writing – original draft, Project administration. **Fatima Noor:** Writing – original draft. **Aram Salehi:** Writing – review & editing, Supervision. **Bassem Al Hariri:** Writing – review & editing, Supervision.

## Declaration of competing interest

The authors declare that they have no known competing financial interests or personal relationships that could have appeared to influence the work reported in this paper.
